# Spore powder of *Ganoderma lucidum* for Alzheimer's disease: A protocol for systematic review

**DOI:** 10.1097/MD.0000000000014382

**Published:** 2019-02-01

**Authors:** Li-Hong Qin, Chen Wang, Li-Wei Qin, Yan-Feng Liang, Guo-Hui Wang

**Affiliations:** a First Ward of Neurology Department; b Second Ward of Neurology Department; c Department of Physical Diagnosis, First Affiliated Hospital of Jiamusi University; d Department of Pathophysiology, Jiamusi University School of Basic Medicine, Jiamusi, China.

**Keywords:** Alzheimer's disease, efficacy, safety, spore powder of *Ganoderma lucidum*, systematic review

## Abstract

**Background:**

Previous studies have reported that spore powder of *Ganoderma lucidum* (SPGL) may be effective for the treatment of Alzheimer's disease (AD). However, its efficacy is still inconclusive. Thus, this systematic review will aim to assess its efficacy and safety for AD.

**Methods:**

We will search the electronic databases of Cochrane Central Register of Controlled Trials, EMBASE, MEDILINE, the Cumulative Index to Nursing and Allied Health Literature, Allied and Complementary Medicine Database, and Chinese Biomedical Literature Database to assess the efficacy and safety of SPGL for patients with AD from their inceptions to the present. All case–control studies and randomized controlled trials will be considered for inclusion in this study. Two review authors will independently perform the study selection, data extraction, and risk of bias evaluation.

**Results:**

The primary outcome includes the cognitive status for patients. The secondary outcomes consist of the quality of life, AD symptoms, and adverse events.

**Conclusions:**

This systematic review will present the existing evidence for the efficacy and safety of SPGL for treating patients with AD.

**Dissemination and ethics:**

The results of this systematic review will be disseminated by through peer-reviewed journals. It does not needs ethic approval, because it does not involve individual patient data.

**Systematic review registration:**

PROSPERO CRD42019119426.

## 1 Introduction

Alzheimer's disease (AD) is a very common chronic progressive neurodegenerative disorder characterized by cognitive dysfunction in elderly.^[1–3]^ It is clinically characterized by loss of reasoning, memory, language, and accompanied by varying degrees of personality changes.^[4–6]^ It has been reported that such condition is the most reason of dementia, contributing for 60% to 70% in patients with dementia.^[7–9]^ Presently, about 47 million patients are diagnosed with dementia worldwide, and this number may increase triple by 2050.^[10–11]^ Thus, the huge amount cost for this disorder brings burdens and pressures to both families and society.^[12]^

There exists no effective treatment for this disorder. Currently, several managements are available for the treatment of AD, and they primarily aimed at cognition improvement and symptoms relief.^[13–16]^ These treatments mainly include pharmacotherapy and physical exercise.^[14–15]^ However, they can only relieve symptoms and also accompanied lots of side effects, especially for medications.^[17]^

Alternative treatment such as traditional Chinese herbal medicine, including spore powder of *Ganoderma lucidum* (SPGL), and acupuncture has also reported to treat AD effectively, especially for SPGL.^[18–20]^ However, no systematic review assesses its efficacy and safety, and so its efficacy is still unclear. Thus, it is very necessary to perform a systematic review to evaluate the efficacy and safety of SPGL for the treatment of AD.

## 2 Methods

### 2.1 Objective

This systematic review aims to evaluate the efficacy and safety of SPGL for patients with AD.

### 2.2 Study registration

This protocol review has been registered in PROSPERO with CRD42019119426.

### 2.3 Inclusion criteria for study selection

#### 2.3.1 Types of studies

All eligible randomized controlled trials (RCTs) and case–control studies will be considered for the assessment of the efficacy and safety of SPGL for AD without any language or publication restrictions. However, the non-RCTs, case reports, case series, uncontrolled trials, and laboratory studies will be excluded.

#### 2.3.2 Types of patients

Adult patients who are clinically diagnosed with AD will be considered in this study. However, patients who have other disorders that may affect cognitive function will be excluded, such as stroke, cancer, Parkinson disease, and traumatic brain injury. Additionally, patients will also be excluded if they had Lewy body, frontotemporal, and vascular dementia, or any other rare forms of dementia, except AD.

#### 2.3.3 Types of interventions

Any types of SPGL treatment will be considered. However, if it combined with other therapies, it will be not included. Control therapy may consist of any other interventions, except the SPGL.

#### 2.3.4 Types of outcome measurements

The primary outcome is cognitive status during the study period, as measured by the AD assessment scale-cognitive or other related scales. The secondary outcomes are quality of life, as assessed by the World Health Organization Quality of Life questionnaire (WHOQOL-BREF) or other related scales; AD symptoms, as evaluated by the neuropsychiatric index, and other associated scales; as well as the adverse events.

### 2.4 Search methods for the identification of studies

#### 2.4.1 Search electronic bibliographic databases

We will search the following electronic bibliographic databases from their inceptions to the present for any potential trials. These databases consist of Cochrane Central Register of Controlled Trials (CENTRAL), EMBASE, MEDLINE, the Cumulative Index to Nursing and Allied Health Literature, the Allied and Complementary Medicine Database, and Chinese databases of Chinese Biomedical Literature Database. Strategy details for CENTRAL are presented in Table 1. Any other electronic databases will be also searched by the similar strategies.

**Table 1:**
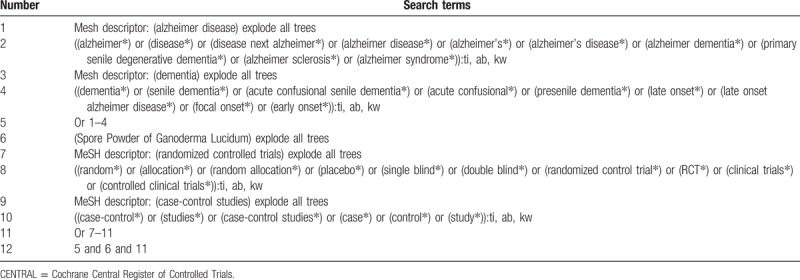
Search strategy applied in CENTRAL database.

#### 2.4.2 Search for other resources

A manual search will also be carried out to search the associated references lists of included trials and relevant reviews, as well as the websites of clinical registrations to avoiding missing any potential studies.

### 2.5 Data collection and analysis

#### 2.5.1 Study selection

**Figure 1:**
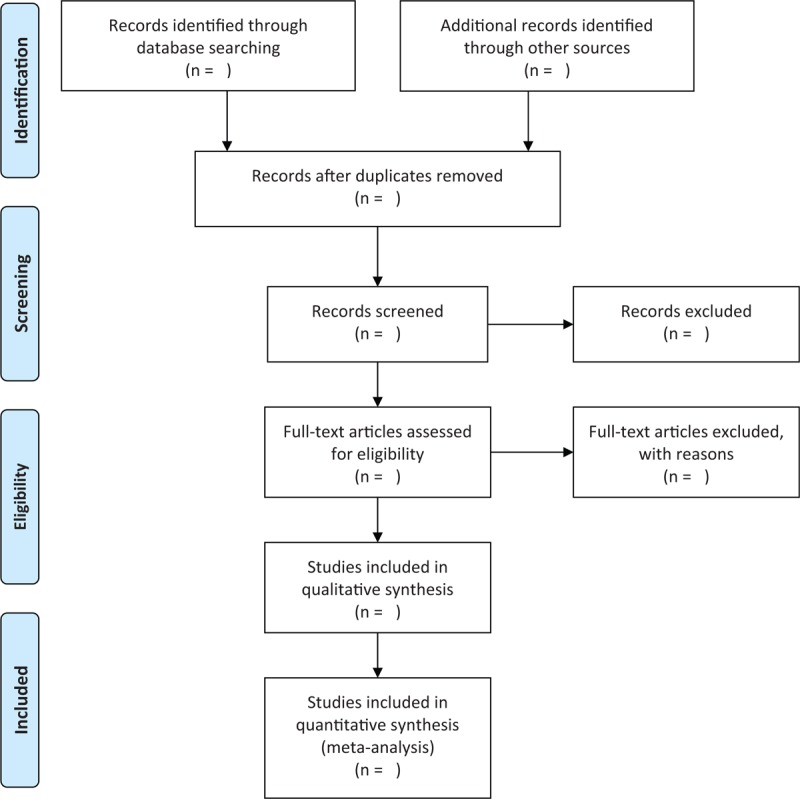
Flowchart of study selection.

Two review authors will independently select the potentially eligible studies based on the predefined eligibility criteria. Any diversity between 2 authors will be resolved by a third involved through discussion. All procedures of study selection will be performed according to the PRISMA flow chart and its results will be shown in Figure 1.

#### 2.5.2 Data extraction and management

A predefined standard data collection form will be utilized to extract data by 2 independent review authors. The form will consist of general information (such as authors, title, publication year, and location), study methods (such as study design, sample size, details of randomization, blinding, allocation, incomplete or selecting report, or any other bias information), participants (such as interventions of different groups), and outcomes (including primary, secondary, and safety outcomes). If divergences will occur between 2 review authors regarding the data extraction, a third review author will be invited to resolve the matter by discussion.

#### 2.5.3 Risk of bias in included studies

The risk of bias in each included study will be conducted by 2 independent review authors using the Cochrane Risk of Bias Tool. The tool includes 7 aspects and each domain will be assessed as high, unclear, and low risk of bias. Any dissimilarity will be resolved by consensus with a third review author.

#### 2.5.4 Measurement of treatment effect

RevMan 5.3 software and STATA 12.0 software will be used to analyze the data. For dichotomous data, the pooled results will be presented as risk ratio with 95% confidence intervals (CIs). For continuous data, the pooled results will be summarized as the mean difference (MD) or standardized MD with 95% CIs.

#### 2.5.5 Unit of analysis

If the eligible cross-over studies will be included, the data of first study period only will be analyzed to avoid the carryover effects.

#### 2.5.6 Dealing with missing data

Any missing, or insufficient or unclear data will be contacted the original trial corresponding authors to request those data. If those data will not be obtainable, only the remaining available will be analyzed, and then it will be discussed as a major limitation.

#### 2.5.7 Subgroup analysis

Subgroup analysis will be conducted to identify any substantial heterogeneity and will be used to investigate the heterogeneity by different treatment types, control therapies, types of diagnostic criteria, treatment duration, and outcome measurements.

#### 2.5.8 Assessment of heterogeneity

The Cochrane *I*^2^ and *Q* statistic tests will be utilized to evaluate the heterogeneity. If the values of *I*^*2*^ > 50%, and/ or *Q* statistic test <0.10 will be identified, the significant heterogeneity will be considered.

#### 2.5.9 Data synthesis

If the values of I^2^ ≤ 50% will be detected, the fixed-effect model will be utilized to pool and to analyze the outcome data. Otherwise, the random-effect model will be applied, and subgroup analysis will also be conducted to identity any potential reasons that may cause the heterogeneity. If significant heterogeneity still exists after the subgroup analysis performance, then the data will not be pooled, and the results will be presented with a narrative summary.

#### 2.5.10 Publication biases

Funnel plot will be conducted to identify any publication bias if there will be more than 10 trials included. In addition, Egg linear regression test and Begg rank correlation test will be operated to detect the funnel plot asymmetry.

#### 2.5.11 Sensitivity analysis

Sensitivity analysis will be applied to detect the stability of the pooled results based on the methodological quality, sample size, and missing data.

## 3 Discussion

Currently, no systematic review and meta-analysis have been conducted regarding the efficacy and safety of SPGL for the treatment of AD. In this systematic review, we will first explore this issue and will search as comprehensive literature as possible without language or publication restrictions to identify more potentially eligible studies. All potential studies regarding the SPGL for the treatment of AD will be fully considered.

This protocol of systematic review will summarize the up-to-date to assess the efficacy and safety of SPGL for the treatment of AD. The findings of this study will provide the evidence to decide whether SPGL will achieve promising benefits in patients with AD. Nevertheless, the safety of SPGL will also be assessed. Additionally, the evidence may also yield helpful evidence for clinical practice, as well as for the health policy-makers.

## Author contributions

**Conceptualization:** Li-Hong Qin, Li-Wei Qin, Guo-Hui Wang.

**Data curation:** Li-Hong Qin, Li-Wei Qin, Yan-Feng Liang, Guo-Hui Wang.

**Formal analysis:** Yan-Feng Liang, Guo-Hui Wang.

**Funding acquisition:** Li-Hong Qin.

**Investigation:** Chen Wang, Li-Wei Qin.

**Methodology:** Yan-Feng Liang, Guo-Hui Wang.

**Project administration:** Li-Wei Qin, Guo-Hui Wang.

**Resources:** Li-Hong Qin, Chen Wang, Yan-Feng Liang.

**Software:** Li-Hong Qin, Yan-Feng Liang, Guo-Hui Wang.

**Supervision:** Chen Wang, Li-Wei Qin, Yan-Feng Liang.

**Validation:** Li-Hong Qin, Chen Wang, Guo-Hui Wang.

**Visualization:** Li-Hong Qin, Chen Wang, Guo-Hui Wang.

**Writing – original draft:** Li-Hong Qin, Chen Wang, Li-Wei Qin, Yan-Feng Liang, Guo-Hui Wang.

**Writing – review and editing:** Li-Hong Qin, Chen Wang, Li-Wei Qin, Yan-Feng Liang, Guo-Hui Wang.
